# The communication role of extracellular vesicles in the osteoarthritis microenvironment

**DOI:** 10.3389/fimmu.2025.1549833

**Published:** 2025-03-17

**Authors:** Pu Chen, Lingfeng Zeng, Ting Wang, Jianbo He, Shuai Xiong, Gang Chen, Qingfu Wang, Haiyun Chen, Jiewei Xie

**Affiliations:** ^1^ The Second Affiliated Hospital of Guangzhou University of Chinese Medicine, Guangdong Provincial Hospital of Chinese Medicine, Guangzhou, China; ^2^ State Key Laboratory of Traditional Chinese Medicine Syndrome, The Second Affiliated Hospital of Guangzhou University of Chinese Medicine, Guangdong Provincial Hospital of Chinese Medicine, Guangzhou, China; ^3^ Guangdong Provincial Key Laboratory of Chinese Medicine for Prevention and Treatment of Refractory Chronic Diseases, The Second Clinical College of Guangzhou University of Chinese Medicine, Guangzhou, China; ^4^ Bone and Joint Research Team of Degeneration and Injury, Guangdong Provincial Academy of Chinese Medical Sciences, Guangzhou, China; ^5^ Department of Operating Room, Ji’an County Traditional Chinese Medicine Hospital, Ji’an, Jiangxi, China; ^6^ School of Anesthesiology, Shandong Second Medical University, Weifang, Shandong, China; ^7^ Department of Orthopedic Surgery, Jiangxi University of Traditional Chinese Medicine Affiliated Hospital, Nanchang, Jiangxi, China; ^8^ Department of Orthopedic Surgery, Beijing University of Chinese Medicine Third Affiliated Hospital, Beijing, China; ^9^ Guangdong Provincial Hospital of Chinese Medicine, Zhuhai Hospital, Zhuhai, China

**Keywords:** osteoarthritis, extracellular vesicles, microenvironment, synovial inflammation, cartilage degeneration

## Abstract

Osteoarthritis (OA) is the most common degenerative joint disease worldwide, characterized by synovial inflammation, cartilage loss, and reactive hyperplasia of subchondral bone, affecting the quality of life of hundreds of millions of people. However, the molecular mechanisms underlying the occurrence and progression of OA remain unclear, and there is no therapy can substantially interrupt or reverse the destructive process of OA. More insight into the pathogenesis of OA may result in innovative therapeutics. The OA microenvironment plays a pivotal role in the development and progression of OA, which encompasses chondrocytes, adipocytes, synovial fibroblasts, endothelial cells, and immune cells. Extracellular vesicles (EVs) have emerged as a novel form of intercellular communication, mediating the transfer of a range of bioactive molecules to create a specific microenvironment. Recent studies have reported that the cargos of EVs play a crucial role in the pathogenesis of OA, including noncoding RNAs (ncRNAs), proteins, and lipids. This review systematically analyzes and summarizes the biological characteristics and functionalities of EVs derived from diverse cellular sources, especially how EVs mediate communication between different cells in the OA microenvironment, with a view to providing new insights into the pathogenesis of OA.

## 1 Introduction

**Figure 1:**
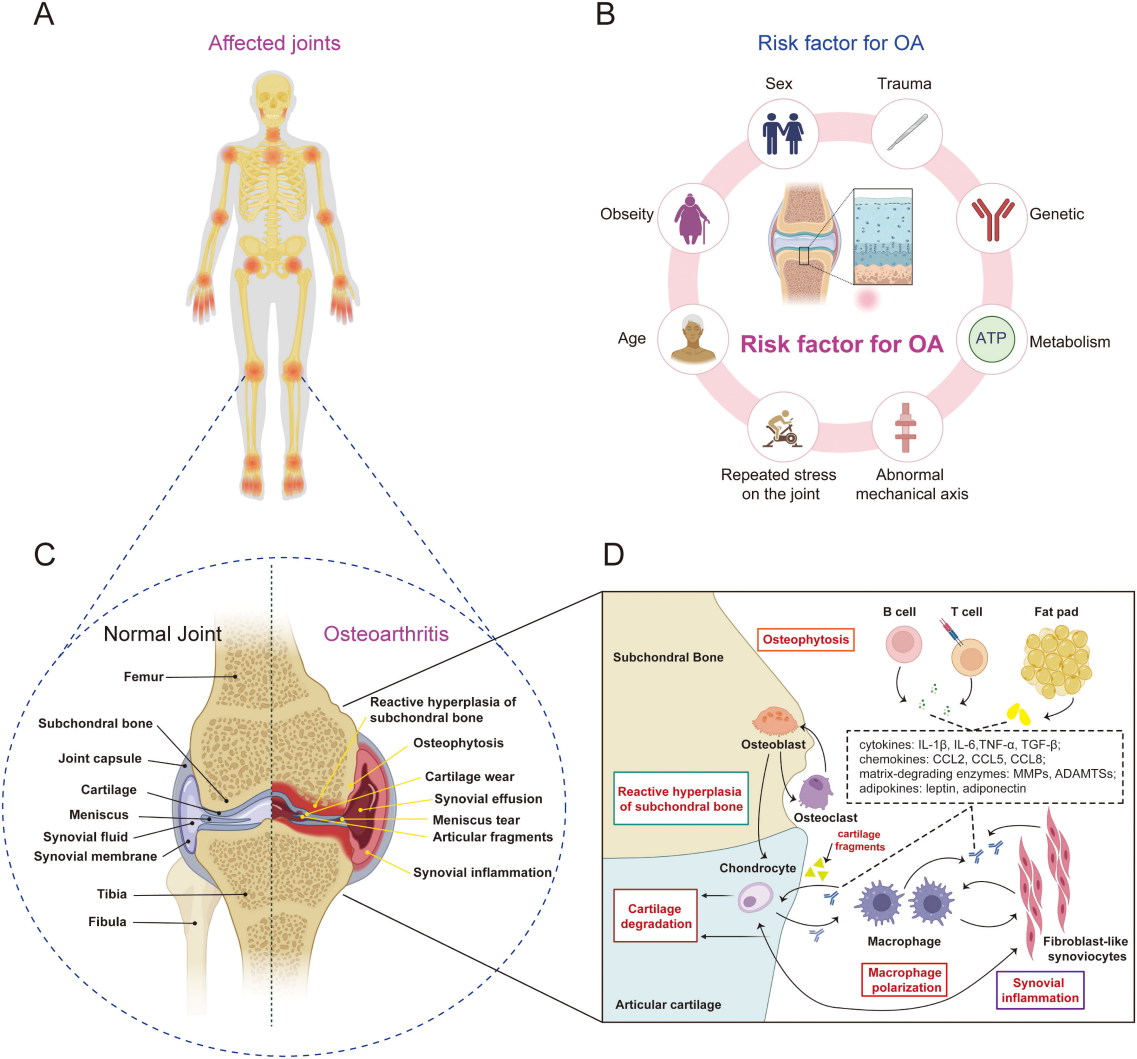
The pathophysiological characteristics of OA. **(A)** OA affects multiple joints throughout the body, such as the temporomandibular joint, shoulder joint, elbow joint, and interphalangeal joints, with the most prevalent being OA of the knee joint. **(B)** the risk factor for OA, include specific systemic risk factors (such as genetics, age, obesity, and sex) as well as mechanical factors (such as trauma, abnormal lower limb alignment, and repeated stress on the joint). **(C, D)** the pathophysiological characteristics of OA include synovial inflammation, progressive cartilage degeneration, abnormal hyperplasia of the subchondral bone, and the formation of osteophytes.

Osteoarthritis (OA) is the most widespread musculoskeletal system disease globally, affecting over 500 million people, accounting for approximately 7.6% of the global population, and exhibiting a rapid growth trend (1, 2) (**Figure 1**). Age, obesity, and sex are the major intrinsic risk factors for OA. OA is distinctively characterized by synovial inflammation, progressive cartilage degeneration, reactive hyperplasia of subchondral bone, and the development of osteophytes, all of which cumulatively exert a detrimental effect on the quality of life of individuals as they advance in age (1, 3). Despite the numerous treatment options available for OA, there is currently no method that can halt or reverse the progression of the disease (4). Joint replacement surgery, considered the gold standard for treating end-stage OA, still faces challenges such as unsatisfactory outcomes, infection risks, significant trauma, and high costs, among other complications (5, 6). Therefore, there is an urgent need to further delve into the pathogenesis of OA in order to aid in the discovery of novel treatment modalities, slow down the progression of OA, prevent early- to mid-stage OA from rapidly advancing to the late stage, and ultimately offer patients better treatment options and an improved quality of life.

It has been traditionally believed that OA is a low inflammatory disease driven by cartilage damage (7–9). Under abnormal mechanical stress, cartilage wear occurs, resulting in the production of small cartilage fragments, which activate danger-associated molecular patterns (DAMPs) and promote the activation of synovial macrophages, triggering synovial inflammation (10). In another aspect, accumulating evidence suggests that synovial inflammation may be a driver in the pathological progression of OA (11–14). Atukorala et al. discovered that in the course of knee OA, synovitis consistently precedes cartilage degeneration, implying that synovitis serves as a precursor for OA (12). However, both theories agree that the disruption of the intra-articular microenvironment is an indispensable condition for the progression of OA. The intra-articular microenvironment is composed of various cells, such as chondrocytes, adipocytes, synovial fibroblasts, synovial macrophages, osteoblasts, osteoclasts (15–18). These cells secrete various metabolic factors and inflammatory factors through paracrine, autocrine, and endocrine pathways, interacting with each other to maintain the homeostasis of articular cartilage. OA severely disrupts this microenvironment. Activated synovial macrophages release substantial amounts of inflammatory cytokines and chemokines, including interleukin-1β (IL-1β), IL-6, tumor necrosis factor-α (TNF-α), and transforming growth factor-beta (TGF-β), which contribute to the establishment of an inflammatory microenvironment (19). This microenvironment subsequently prompts chondrocytes to excessively secrete protease matrix-degrading enzymes, encompassing matrix metalloproteinases (MMPs) and disintegrin and metalloproteinase with thrombospondin motifs (ADAMTSs). This process leads to the degradation of the extracellular matrix (ECM), thereby accelerating cartilage damage and triggering the progression of OA (20, 21). Nevertheless, the exact mechanisms of how these cells communicate, transmit signals, and influence the progression of OA are still unclear.

**Figure 2:**
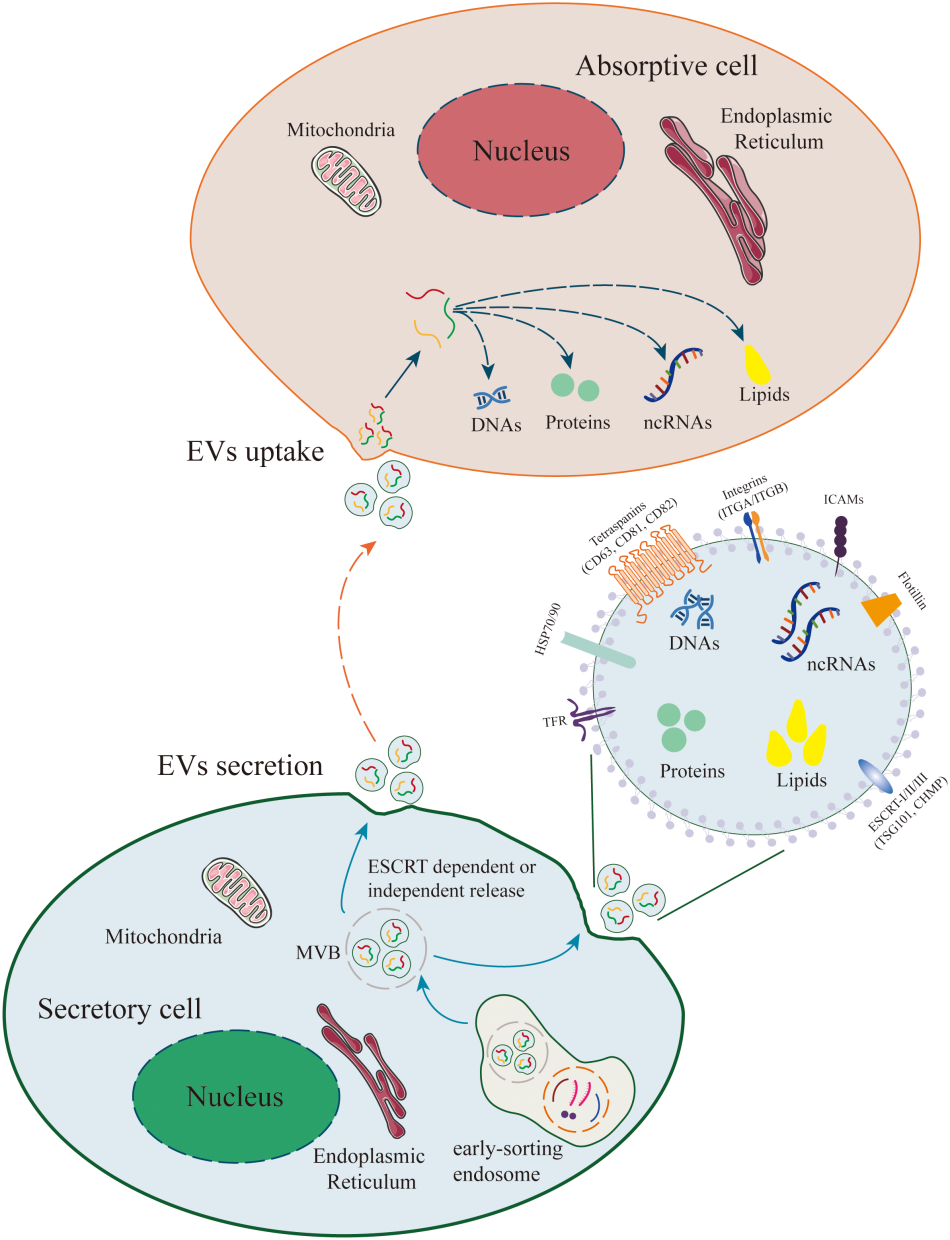
Release and Uptake of Extracellular Vesicles (EVs). The biogenesis of EVs originates from plasma membrane endocytosis, which includes cell surface proteins, extracellular soluble proteins, and other small molecules, resulting in the formation of early sorting endosomes (ESE). Early endosomes mature into multivesicular bodies (MVBs) and form intraluminal vesicles (ILVs, i.e., EVs) within MVBs through ESCRT-dependent or ESCRT-independent pathways. MVBs fuse with the plasma membrane for EVs release. EVs can contain a wide range of molecules, including proteins, metabolites, lipids, DNA, and RNAs (mRNA, non-coding RNAs). In recipient cells, EVs can activate intercellular signaling through receptor-ligand interactions. Additionally, EVs can be internalized via endocytosis or direct fusion and release their cargo into recipient cells, thereby mediating various physiological and pathological processes.

Extracellular vesicles (EVs) have attracted widespread attention in the past two decades due to their characteristics such as being secreted by virtually all cells and being able to serve as messengers between cells (22, 23) (**Figure 2**). Due to their lipid-enclosed structures, EVs are capable of carrying specific proteins, miRNAs, lncRNAs, circRNAs, and other growth factors, while effectively shielding them from enzymatic degradation in complex microenvironments. Consequently, EVs play a pivotal and indispensable role in intercellular communication, as they facilitate the transmission of information and influence, as well as regulate, diverse biological processes within target cells. EVs originating from various cell types, such as osteoclasts, osteoblasts, chondrocytes, adipocytes, synovial fibroblasts, and immune cells, play crucial regulatory roles in the intra-articular microenvironment (24, 25). These EVs, released into the synovial fluid, can influence and regulate biological processes of surrounding or distant cells, serving as a key factor in maintaining the homeostasis of the intra-articular microenvironment. Gaining a deeper understanding of the roles of EVs derived from various cell types in inflammatory microenvironments will contribute significantly to our comprehension of the biological mechanisms underlying the development and progression of OA. Additionally, it may facilitate the discovery of novel therapeutic targets for OA, with the potential to halt and even reverse the progression of the disease. In this review, we focus our attention on the role of EVs from various cell types in OA pathology and the opportunities they present for novel OA treatment modalities. The search strategy is attached in Appendix 1. The roles of EVs from different cellular sources and tissues in the pathogenesis of OA were listed in **Table 1**.

**Table 1:** The roles of EVs from different cellular sources and tissues in the pathogenesis of OA.

EVs cargos	EVs-derived cells/tissue	EVs-accepted cells	Biological effects on OA	Refs
MGP, TNAP, NPP1	chondrocytes	chondrocytes	Under abnormal biomechanical stimulation, chondrocytes secrete more EVs, which promote the expression of MMP-13, RUNX2, leading to the degradation and calcification of chondrocytes.	(26)
miR-140, miR-107	chondroprogenitor cells	chondrocytes	OA chondroprogenitor cells derived EVs have a high expression of miR-140 and miR-107, respectively delay cartilage damage by inducing the expression of chondrocyte anabolic genes and inhibiting the expression of chondrocyte catabolic genes	(27)
calcium and phosphorus	chondrocytes	chondrocytes	HDAC6 promotes the secretion of autophagic LC3-positive calcified EVs from chondrocytes, inducing pathological cartilage calcification	(28)
/	chondrocytes	chondrocytes, synovial macrophages, osteoblast	Inflammatory C-EVs can enhance hyaline cartilage thickness, reduce synovial inflammation, and promote subchondral bone formation in the early stage of OA	(29)
miR-95-5p	chondrocytes	chondrocytes	Inflammatory C-EVs have low expression of miR-95-5p, while overexpression of miR-95-5p targets and inhibits the expression of HDAC2/8, maintaining the stability of the ECM and promoting cartilage formation.	(30)
lncRNA PVT1	chondrocytes	chondrocytes	C-EVs PVT1 can sponge miR-93-5p, thereby regulating the expression of HMGB1, which in turn affects the TLR4/NF-κB signaling pathway, promoting cartilage inflammation and degradation	(31)
LOC102546541	chondrocytes	chondrocytes	C-EVs highly expressed LOC102546541, downregulated miR-632, enhanced the function of MMP13, thereby accelerating the degradation of cartilage	(32)
circ-BRWD1	chondrocytes	chondrocytes	OA C-EVs circ-BRWD1 acts as a sponge for miR-1277, which in turn positively regulates the expression of TRAF6, promoting cartilage damage.	(36)
circ_0001846	chondrocytes	chondrocytes	OA C-EVs circ_0001846 acts as a sponge for miR-149-5p, which in turn positively regulates the expression of WNT5B, promoting chondrocyte apoptosis, inflammation, and ECM degradation.	(37)
circ-PRKCH	chondrocytes	chondrocytes	OA C-EVs circ-PRKCH acts as a sponge for miR-502-5p, which in turn positively regulates the expression of ADAMTS5, promoting chondrocyte apoptosis, inflammation, and inhibiting proliferation and migration.	(38)
Cx43	chondrocytes	chondrocytes, synovial cells, osteoblasts	OA C-EVs contain high levels of Cx43, which can induce senescence in chondrocytes, synovial cells, and osteoblasts.	(39)
miR-9-5p	chondrocytes	osteoblasts	Chondrocytes under mechanical strain can secrete EVs containing miR-9-5p, taken up by osteoblasts, inhibit osteoblast differentiation by targeting the suppression of KLF5, thus slowing down the sclerosis of subchondral bone.	(40)
miR-199a-5p, miR-339-5p, miR-25-3p, miR-186-5p	chondrocytes	osteoblasts	chondrocytes can alter the expression levels of certain miRNAs in their EVs under stress stimuli which enhances the expression of RUNX2 and SOX9 in osteoblasts, facilitating osteogenesis and promoting the progression of subchondral bone sclerosis	(41)
miR-221-3p	chondrocytes	osteoblasts	OA C-EVs miR-221-3p inhibited the expression of CDKN1B/p27, TIMP-3, Tcf7l2/TCF4, and ARNT, which subsequently suppresses the bone-forming capacity of osteoblasts.	(42)
miR-125	chondrocytes	osteoblasts	OA C-EVs transfer miR-125 to osteoblasts, disrupting the homeostasis of subchondral bone and exacerbating cartilage damage in aging mice.	(43)
/	chondrocytes	synovial macrophages	Primary chondrocyte-derived EVs can promote the infiltration of M2 anti-inflammatory macrophages while reducing the number of M1 pro-inflammatory macrophages, regulating the immune response within the joint.	(44)
miR-449a-5p	chondrocytes	synovial macrophages	OA C-EVs exhibit high expression of miR-449a-5p, which inhibits macrophage autophagy by suppressing ATG4B, thereby promote the activation of the inflammasome.	(45)
OANCT	chondrocytes	synovial macrophages	EVs derived from functionally impaired chondrocytes exhibit high expression of OANCT, which binds to FTO. They regulate the stability of PIK3R5 mRNA in an m6A modification-dependent manner, thereby inhibiting macrophage autophagy and promoting M1 polarization.	(46)
/	FLSs	FLSs	Aging OA FLSs-EVs induce senescence in non-aging FLSs and promote the formation of an inflammatory phenotype.	(47)
/	FLSs	synovial macrophages	Inflammatory FLSs-EVs promote HIF1A expression in macrophages, thereby upregulating the expression of key enzymes in the glycolysis pathway, including GLUT1, HK2, PKM2, and LDHA. This activation of aerobic glycolysis in macrophages promotes M1 polarization.	(48)
miR-146	FLSs	synovial macrophages	FLSs-EVs highly express miR-146, which inhibits the TLR4/TRAF6/NF-κB signaling pathway by targeting TRAF6, inducing the polarization of synovial macrophages from the M1 to M2 type.	(49)
/	FLSs	chondrocytes	EVs from IL-1β - stimulated FLSs significantly promote cartilage degradation.	(50)
miR-126-3p	FLSs	chondrocytes	EVs from FLSs that overexpress miR-126-3p can promote the migration and proliferation of chondrocytes, inhibit apoptosis and the expression of inflammatory factors.	(51)
miR-150-3p	FLSs	chondrocytes	Normal FLS-EVs highly express miR-150-3p, which inhibits the Trim14/NF-κB/IFNβ axis, suppresses chondrocyte inflammation, and delays the progression of OA.	(52)
miR-214-3p	FLSs	chondrocytes	EVs secreted by FLSs with overexpressed miR-214-3p can promote the proliferation of chondrocytes, inhibit apoptosis, and reduce the levels of inflammatory cytokine	(53)
miR-4738-3p	FLSs	chondrocytes	Normal FLS-EVs highly express miR-4738-3p, which inhibits chondrocyte inflammation and delays cartilage damage by suppressing COL2A1 expression	(54)
PCGEM1	FLSs	chondrocytes	OA FLSs-EVs highly express PCGEM1, PCGEM1 acts as a molecular sponge for mi-142-5p, leading to the upregulation of RUNX2, then promotes chondrocyte apoptosis and the degradation of cartilage matrix.	(55)
H19	FLSs	chondrocytes	FLSs derived EVs highly express H19, regulate miR-106b-5p to promote the expression of TIMP2, then inhibit the activity of MMPs, thereby inhibiting the degradation of cartilage matrix in OA.	(56)
miR-25-3p	FLSs	chondrocytes	FLS-EVs miR-25-3p inhibited the transcription of CPEB1, thereby alleviating pyroptosis of chondrocytes.	(60)
miR-19b-3p	FLSs	chondrocytes	OA FLSs derived EVs carry miR-19b-3p and deliver it to chondrocytes, targeting and inhibiting the SLC7A11, thus promoting the ferroptosis and damage of chondrocytes.	(65)
miR-1246	M1 macrophages	chondrocytes	miR-1246 has a high expression level in M1-EVs, inhibits the expression of GSK3β and Axin2, thereby activating the Wnt/β-catenin signaling pathway and leading to inflammation in chondrocytes	(66)
miR-146b-5p	M1 macrophages	chondrocytes	M1-EVs highly express miR-146b-5p, which targets and inhibits USP3 and SOX5 in chondrocytes, promoting cartilage degradation.	(67)
/	M1 macrophages	chondrocytes	M1-EVs activate caspase-11, which subsequently activates GSDMD, leading to pyroptosis in chondrocytes.	(68)
miR-363	M1 macrophages	chondrocytes	M1-EVs highly express miR-363, and inhibit chondrocyte proliferation and survival and induce the expression of inflammatory genes through G3BP2	(69)
miR-26b-5p	M2 macrophages	synovial macrophages, chondrocytes	The expression of miR-26b-5p is significantly increased in M2-EVs. Targeting the TLR3 signaling pathway, it can re-polarize M1 macrophages into M2 phenotype. It can also inhibit inflammation-induced chondrocyte hypertrophy by targeting COL10A1.	(70)
/	M2 macrophages	synovial macrophages	M2-EVs can reprogram M1 macrophages into the M2 phenotype by NLR, TNF, NF-κB, and TLR signaling pathways	(71)
miR-21-5p	M2 macrophages	synovial macrophages, chondrocytes	M2-EVs can reprogram M1 macrophages into the M2 phenotype via miR-21-5p, and then prevent articular cartilage damage	(72)
/	M2 macrophages	KOA rat model	Intravenous injection of M2-EVs into the tail vein of rats with KOA can reduce the joint tissues inflammation and improve the structure of cartilage tissue.	(73)
miR-210-5p	osteoblasts	chondrocytes	miR-210-5p is highly enriched in the EVs of osteoblasts in the sclerotic subchondral bone of OA patients. It enhances the expression of MMP13, ADAMTS5, RUNX2, and COL10 in articular chondrocytes, while inhibiting the expression of SOX9 and COL2A1.	(75)
miR-212-3p	osteoclasts	chondrocytes	osteoclast-derived EVs highly express miR-212-3p, which is absorbed by chondrocytes and targets the suppression of Smad2, accelerating cartilage matrix degradation in OA	(76)
miR-21a-5p, miR-214-3, miR-148a-3p, miR-199a-3p, miR-378a-3p, miR-30, miR-200, miR-29	osteoclasts	chondrocytes	The highly expressed miRNA transferred from osteoclast-derived EVs to chondrocytes suppresses the expression of TIMP-2 and TIMP-3, thereby promoting cartilage matrix degradation.	(77)
SOX9	monocytes	chondrocytes	Monocytes transfer SOX9 to chondrocytes via EVs, increasing the expression of COL1A2, ACAN, proteoglycans and type II collagen, thus facilitating cartilage repair.	(78)
/	neutrophil	FLSs	EVs derived from healthy neutrophils can downregulate the expression of TNFα-induced IL-5, IL-6, IL-8, MCP-1, IFN-γ, and MIP-1β in FLS.	(79)
/	neutrophil	chondrocytes	TGF-β-intervened neutrophil EVs significantly promoted the upregulation of SFRP5 expression in chondrocytes, inhibiting the TNF and NF-κB signaling pathways, reducing the production of inflammatory and catabolic factors, and protecting cartilage from damage	(80)
/	vascular endothelial cells	chondrocytes	EVs secreted by vascular endothelial cells can be absorbed by chondrocytes, increasing the ROS content of chondrocytes, exacerbating apoptosis, and inhibiting chondrocyte autophagy.	(81)
/	Platelet	chondrocytes	Platelet-derived EVs regulate the differential expression of 1797 genes in chondrocytes (with 923 genes upregulated and 874 genes downregulated), promote the proliferation and migration of chondrocytes, and significantly promote cartilage regeneration.	(82)
/	Platelet-rich plasma	chondrocytes	EVs derived from Platelet-rich plasma can promote the proliferation and migration of chondrocytes, reduce cell apoptosis, and reverse the activation of the Wnt/β-catenin signaling pathway in chondrocytes	(83)
/	synovial fluid	chondrocytes	synovial fluid EVs from the advanced OA group notably promoted chondrocyte inflammation and inhibited chondrocyte proliferation, thereby accelerating joint degeneration.	(88)
/	synovial fluid	monocytes	SF-EVs induce monocytes to release the anti-inflammatory cytokine IL-1Ra, thereby regulating the immune microenvironment in OA	(89)
/	synovial fluid	chondrocytes	OAs SF-EVs significantly increase the inflammatory response of chondrocytes, inhibit chondrocyte proliferation, and thus promote joint degeneration.	(90)
miR-182-5p	synovial fluid	chondrocytes	The expression of miR-182-5p is significantly reduced in OA SF-EVs. Overexpression of miR-182-5p can inhibit the expression of TNFAIP8, thereby inhibiting autophagy and apoptosis of chondrocytes through ATG3.	(91)
/	synovial fluid	synovial macrophages	OA SF-EVs can stimulate the polarization of macrophages into the M1 phenotype, leading to the release of various inflammatory factors, chemokines, and MMPs.	(92)
/	synovial tissue	chondrocytes	OA synovial tissue derived EVs significantly inhibited chondrocyte proliferation, promoted apoptosis, and promoted cartilage inflammation and degradation via the NF-κB signaling pathway.	(95)
miR-182	synovial tissue	synovial cells	miR-182 was significantly overexpressed in OA synovial tissue derived EVs, and miR-182 regulates the expression of inflammatory factors by suppressing FOXO3.	(96)
let-7b-5p, let-7c-5p	infrapatellar fat pad	chondrocytes	The high content of let-7b-5p and let-7c-5p in infrapatellar fat pad derived EVs can target and inhibit the expression of LBR, promote ECM degradation, and induce chondrocyte senescence.	(97)

“/” indicates that the article does not describe which cargo in EVs mediates the research mechanism. C-EVs, chondrocytes derived EVs; FLSs-EVs, FLSs derived EVs; M1-EVs, M1 macrophages derived EVs; M2-EVs, M2 macrophages derived EVs; SF-EVs, synovial fluid derived EVs.

## 2 The role of chondrocyte derived EVs in the pathogenesis of OA

**Figure 3:**
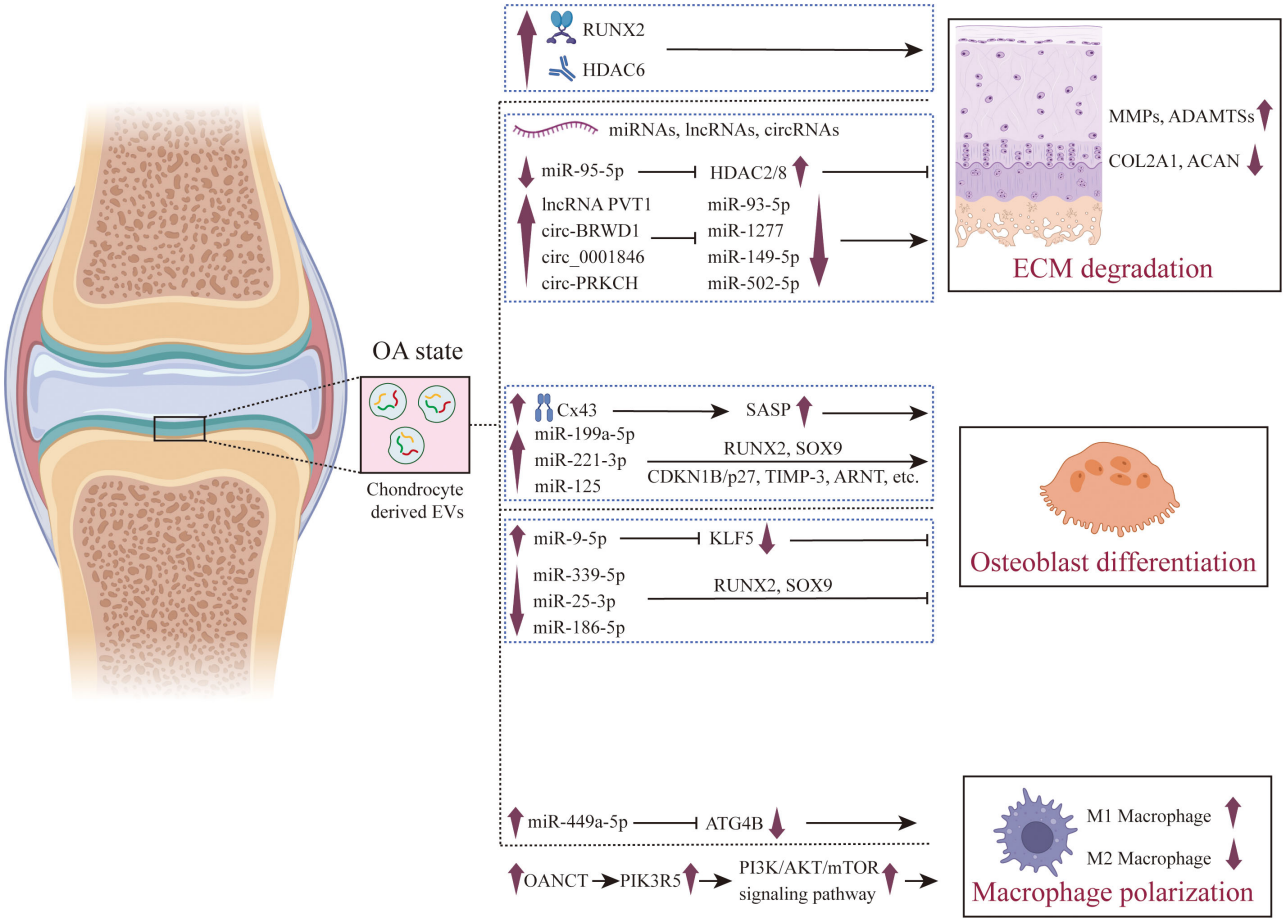
The role of chondrocyte derived EVs in the pathogenesis of OA. The pivotal role of chondrocyte-derived EVs in the pathogenesis of OA is multifaceted. In the context of OA, these EVs are instrumental in the promotion of ECM degradation, which results in cartilage damage. These EVs promote the expression of RUNX2 and HDAC6, as well as the expression of long-noncoding RNAs such as lncRNA PVT1 and circ-PRKCH, and inhibit the expression of miRNAs such as miR-93-5p and miR-502-5p, thereby promoting the high expression of ECM-degrading enzymes (MMPs and ADAMTSs) and inhibiting the expression of genes related to cartilage matrix synthesis in chondrocytes (such as COL2A1 and ACAN). Furthermore, they exert regulatory influence over osteoblasts, guiding them towards osteogenic differentiation, thus playing a role in the process of subchondral bone hardening. These EVs can induce osteogenic differentiation by promoting Cx43 and secreting SASP factors, and can also promote osteogenic differentiation by regulating the expression of miRNAs. Additionally, their regulatory effects on synovial macrophages are crucial in fostering an inflammatory microenvironment conducive to the progression of OA. Chondrocyte derived EVs promote the production of ROS in macrophage mitochondria by up - regulating the expression of miR-449a-5p and lncRNA OANCT, thereby promoting M1 polarization.

Cartilage is a soft connective tissue that lacks a nervous, vascular, and lymphatic system. It is widely present in the joints of the body, performing crucial functions such as providing bodily support, cushioning joint pressure, and absorbing impact during movement. Cartilage is composed of 3% to 5% chondrocytes, the sole cell type in cartilage, and 95% of extracellular matrix (17). Meanwhile, upon cartilage damage, chondrocytes release elevated levels of MMPs and ADAMTSs, which contribute significantly to the reduction and degradation of the ECM. This, in turn, exacerbates cartilage tissue damage, fosters synovial inflammation, disrupts subchondral bone homeostasis, and ultimately culminates in the development of OA (43, 45). During this complex physiological and pathological process, EVs serve as essential messengers, assuming the role of conveying critical signals between chondrocytes and other cells (**Figure 3**).

### 2.1 The role of chondrocyte derived EVs on chondrocytes and ECM

The maintenance of cartilage integrity heavily relies on the integrity and functional status of the cartilage ECM, while under abnormal conditions, chondrocytes can secrete EVs that promote ECM degradation, induce chondrocyte inflammation, and facilitate chondrocyte calcification. Under abnormal biomechanical stimulation, chondrocytes secrete more EVs, which promote the expression of MMP-13, Runt-related transcription factor 2 (RUNX2), and other factors, leading to the degradation and calcification of chondrocytes. Additionally, EVs enhance the expression of nucleotide pyrophosphatase/phosphodiesterase-1 (NPP1) and tissue-nonspecific alkaline phosphatase (TNAP), both of which are key factors in promoting the calcification process. GW4869, as an inhibitor of EVs formation, blocks the generation of EVs, effectively reducing the intercellular transmission of these bioactive molecules. This inhibition decreases the expression levels of genes such as MMP-13, RUNX2, TNAP, and NPP1, thereby helping to restore the normal physiological state of chondrocytes and slowing down the degradation and abnormal calcification of cartilage (26). Ganesh et al. found that there is a large number of differentially expressed miRNAs in OA chondroprogenitor cells derived EVs (CPC-EVs), among which miR-140 induces the expression of chondrocyte synthetic genes, while miR-107 inhibits the expression of chondrocyte degradation genes, suggesting the potential therapeutic role of chondroprogenitor cells in OA (27). Yan and colleagues (28) found that the enhanced activity of histone deacetylase 6 (HDAC6) leads to the deacetylation of microtubule proteins, which affects the stability of microtubules and disrupts the fusion of autophagosomes with lysosomes. When the autophagy flux is obstructed, autophagosomes cannot fuse with lysosomes normally, leading to the accumulation of autophagosomes that are eventually released outside the cell through an atypical secretion pathway, forming EVs. These EVs carry minerals such as calcium and phosphorus, which deposit in the extracellular matrix and promote the formation of calcified nodules. These calcified nodules further affect the structure and function of chondrocytes, leading to cartilage degeneration and pathological calcification of cartilage. By inhibiting the activity of HDAC6, the autophagy flux can be restored, reducing the release of LC3-positive calcified EVs, thus providing a new strategy for the treatment of osteoarthritis. However, Xu found that in the early stage of OA, EVs from inflammatory chondrocytes promote M2 macrophage polarization, while reducing M1 polarization, alleviating synovial inflammation, and increasing the thickness of hyaline cartilage and the content of proteoglycans (29). This research reveals that EVs from inflammatory cartilage play a bidirectional role in the progression of OA, and their underlying mechanisms require further investigation.

### 2.2 The role of chondrocyte derived EVs ncRNAs on chondrocytes and ECM

EVs-derived ncRNAs from chondrocytes play a crucial role in the homeostasis of chondrocytes. The study found that miR-95-5p is significantly down-expressed in the EVs of chondrocytes from patients with OA, while the expression of HDAC2/8 is up-regulated. Moreover, EVs miR-95-5p from chondrocytes can directly target the 3’ untranslated region of HDAC2/8, inhibit their expression, which in turn promotes the maintenance of chondrocyte extracellular matrix homeostasis and the formation of cartilage (30). Meng and colleagues (31) found that the lncRNA PVT1 is upregulated in the serum and chondrocytes of patients with OA, and knockdown of PVT1 can reverse the decreased chondrocyte activity, increased apoptosis, inflammatory response, and collagen degradation induced by lipopolysaccharide (LPS). The underlying mechanism is that EVs PVT1 can sponge miR-93-5p, further regulating the expression of HMGB1, thereby affecting the TLR4/NF-κB signaling pathway. Lai et al. found that EVs from damaged chondrocytes highly expressed LOC102546541, and by downregulating miR-632, they enhanced the function of MMP13, thereby accelerating the degradation of cartilage (32).

CircRNAs are a class of non-coding RNA molecules characterized by their closed circular structure, playing a diverse range of biological roles within the cell. A notable function of circRNAs is serving as “sponges” for miRNAs, where they can regulate the inhibitory effect of miRNAs on target mRNAs by adsorbing specific miRNA molecules, thus finely tuning gene expression (33, 34). Moreover, circRNAs act as messengers between cells, conveying information through mechanisms such as EVs, participating in intercellular communication and coordination (35). This unique capability positions circRNAs as pivotal players in cellular communication and the gene regulatory network. In comparison with normal cartilage tissues, the expression levels of circ-BRWD1 in OA cartilage tissues and IL-1β-treated CHON-001 cells derived-EVs are increased. Suppressing circ-BRWD1 in chondrocyte EVs can effectively mitigate chondrocyte apoptosis, inflammation, and ECM degradation (36). The underlying mechanism involves circ-BRWD1 acting as a sponge for miR-1277, which in turn positively regulates the expression of TRAF6. The expressions of circ_0001846 (37) and circ-PRKCH (38) are upregulated in OA cartilage tissue and IL-1β-treated chondrocytes-derived EVs. Inhibiting circ_0001846 and circ-PRKCH in chondrocyte EVs can significantly reverse IL-1β-mediated chondrocyte apoptosis, inflammation, and ECM degradation. Mechanistically, circ_0001846 and circ-PRKCH positively regulate the expressions of downstream target genes WNT5B and ADAMTS5 by sponging miR-149-5p and miR-502-5p respectively.

### 2.3 The role of chondrocyte derived EVs on subchondral bone

The subchondral bone is intricately linked with articular cartilage to sustain the joint’s functional load-bearing and movement capabilities. Degradation of the ECM, however, reduces the ability of the subchondral bone and articular cartilage to cushion impacts, leading to an undue mechanical burden on the subchondral bone and disrupting the subtle equilibrium that exists between osteoclasts and osteoblasts, thereby hastening the deterioration of cartilage in OA.

EVs released by chondrocytes from OA patients contain high levels of connexin 43 (Cx43) and are capable of inducing a senescence phenotype in target chondrocytes, synovial cells, and osteoblasts. This process leads to the formation of an inflammatory and degenerative joint environment by secreting senescence-associated secretory phenotype (SASP) molecules, including IL-1β, IL-6, and MMPs, thereby accelerating the progression of OA (39). Chondrocytes under mechanical strain can secrete EVs containing miR-9-5p, taken up by osteoblasts, inhibit osteoblast differentiation by targeting the suppression of KLF5, thus slowing down the sclerosis of subchondral bone. By administering EVs rich in miR-9-5p into the joint cavity of an OA mouse model, the progression of OA induced by destabilization of the medial meniscus (DMM) surgery can be mitigated (40). Meanwhile, chondrocytes can alter the expression levels of certain miRNAs in their EVs under stress stimuli (promoting the expression of miR-199a-5p and reducing the expression of miR-339-5p, miR-25-3p, and miR-186-5p), which enhances the expression of RUNX2 and SOX9 in osteoblasts, facilitating osteogenesis and promoting the progression of subchondral bone sclerosis (41). Furthermore, EVs derived from chondrocytes can deliver miR-221-3p to osteoblasts, inhibiting the expression of CDKN1B/p27, TIMP-3, Tcf7l2/TCF4, and ARNT, which subsequently suppresses the bone-forming capacity of osteoblasts (42). In the natural aging, medial meniscus instability, and load-induced OA mouse models, a significant increase in sympathetic activity (such as the levels of norepinephrine) was observed. Sympathetic nerve excitement promotes the overexpression of miR-125 in chondrocytes and transfers miR-125 to osteoblasts through the secretion of EVs, thereby disrupting the homeostasis of the subchondral bone and exacerbating cartilage damage in aging mice (43).

### 2.4 The role of chondrocyte derived EVs on synovial macrophages

Cartilage debris and degraded ECM components are secreted into the synovial fluid, activating DAMPs and stimulating macrophage-like synovial cells to initiate an inflammatory response, secreting a large number of proinflammatory factors, such as cytokines and chemokines. The release of these pro-inflammatory factors into the synovial fluid further exacerbates cartilage damage and ECM degradation, forming a microenvironment that accelerates OA progression (98). The role of chondrocytes-derived EVs in maintaining the inflammatory network of OA has attracted widespread attention. Compared to chondrocytes cultured under normal conditions, chondrocytes cultured in an inflammatory environment can alter the expression of protein content in their EVs. Primary chondrocyte-derived EVs (D0 EVs) can fuse with damaged mitochondria, restoring their normal structure and function, reducing the production of ROS, and increasing intracellular ATP levels, thereby enhancing the energy metabolism of chondrocytes. Additionally, D0 EVs can promote the infiltration of M2 anti-inflammatory macrophages while reducing the number of M1 pro-inflammatory macrophages, regulating the immune response within the joint, and thus slowing down the progression of OA (44).

Ni and colleagues (45) found compared with non-degraded cartilage, degraded cartilage tissue released a significantly increased number of EVs, which can enter into the synovial tissue and macrophages of the joint. EVs from chondrocytes pre-treated with IL-1β are enriched with miR-449a-5p, which were absorbed by macrophages, reduced the expression of ATG4B in macrophages, and inhibited autophagy of macrophages. The reduction in macrophage autophagy leads to an increase in the production of mitochondrial reactive oxygen species (mitoROS), which further enhances the activation of the inflammasome and the production of IL-1β. EVs derived from dysfunctional chondrocytes (DC-EVs) are capable of inhibiting macrophage autophagy and promoting M1 polarization (46). The fundamental mechanism involves the upregulation of OA non-coding transcript (OANCT), a long non-coding RNA, in dysfunctional chondrocytes. This transcript is then released into macrophages via EVs. Once inside the macrophages, OANCT binds to fat mass and obesity-associated protein (FTO) and modulates the stability of phosphoinositide-3-kinase regulatory subunit 5 (PIK3R5) mRNA in a manner dependent on N6-methyladenosine (m6A) modification. This ultimately affects the PI3K/AKT/mTOR signaling pathway. Animal experiments further validate that DC-EVs significantly contribute to histomorphological changes in cartilage, upregulating the expression of pro-inflammatory factors such as TNF-α, IL-12, and IL-6, while inhibiting the expression of anti-inflammatory factors like IL-10 and TGF-β. These actions promote M1 macrophage polarization. Notably, the knockdown of OANCT significantly mitigates this effect, providing further evidence that DC-EVs exacerbate the progression of osteoarthritis (OA) through the mediation of OANCT.

## 3 The role of synovial cells derived EVs in the pathogenesis of OA

The normal structure of the synovial membrane typically consists of two parts: the inner layer of the synovium (also known as the lining layer) and the outer layer of the synovium (also known as the subcutaneous layer or matrix layer) (99, 100). The inner layer of the synovium is composed of macrophages and fibroblasts, with fibroblasts being the predominant cell type in the healthy synovium. The outer layer of the synovium is composed of a variety of connective tissues, rich in type I collagen, microvessels, as well as lymphatic vessels and nerve fibers, but relatively lacks cells (100). In OA, the number of cells in the synovial lining layer increases, especially the proliferation of synovial macrophages, which leads to the thickening of the lining layer. The function of these cells also shifts from protection and lubrication to participating in inflammatory responses and tissue damage. The polarization state of synovial macrophages changes, and they can transform into M1 type (pro-inflammatory) or M2 type (anti-inflammatory) (101).

### 3.1 The role of FLSs derived EVs on FLSs and synovial macrophages

**Figure 4:**
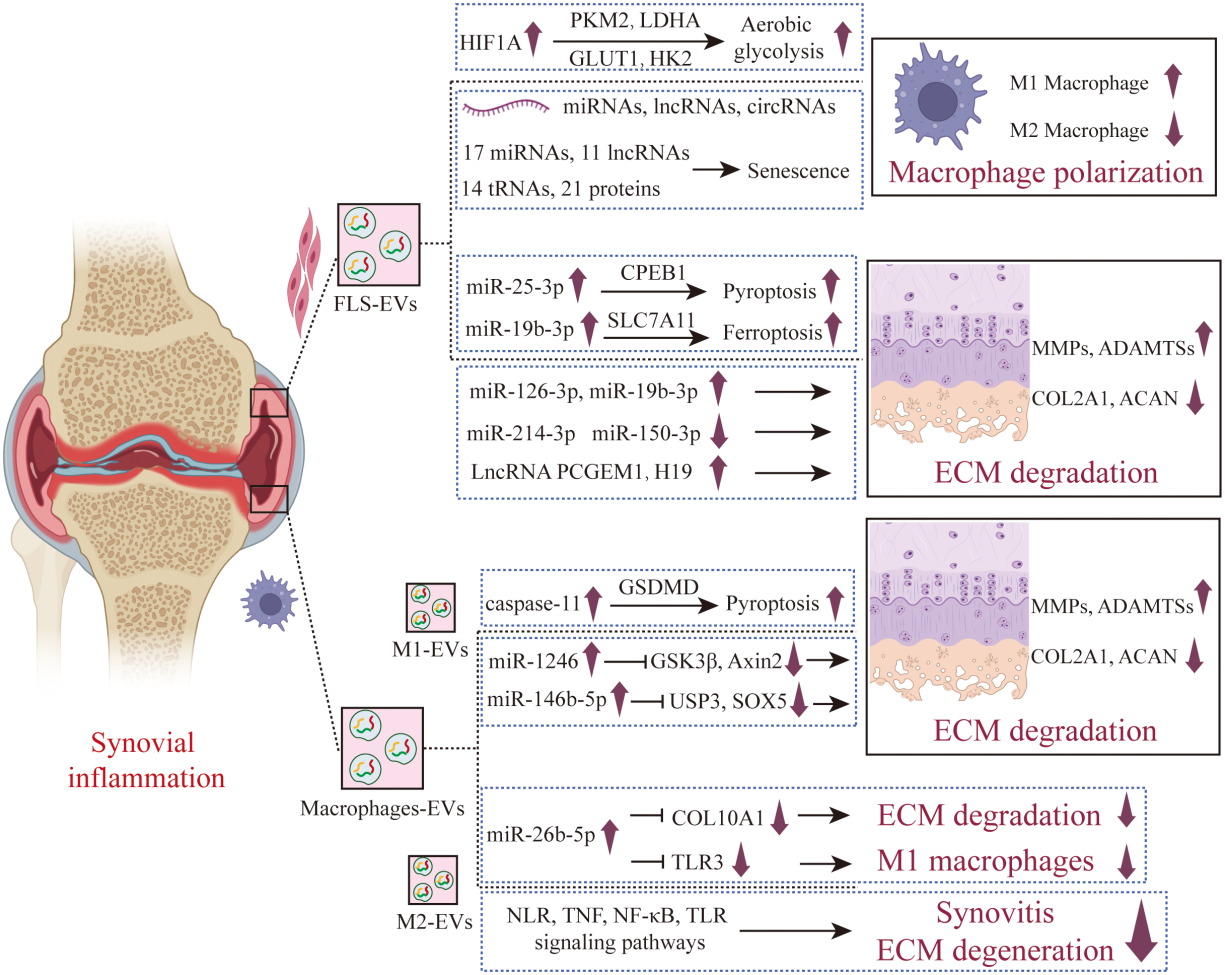
The role of synovial cells derived EVs in the pathogenesis of OA. FLS secreted EVs under OA that promote cartilage inflammation and polarize anti-inflammatory synovial macrophages into pro-inflammatory synovial macrophages. A new type of programmed cell death has also been studied in the regulation of chondrocytes by FLS-EVs, finding that FLS-EVs promote pyroptosis and ferroptosis in chondrocytes. M1-EVs promote cartilage degradation and pyroptosis, while M2-EVs exhibit a protective effect on joints, which has been confirmed *in vivo* assays. FLS-EVs, Fibroblast-like synoviocyte-derived extracellular vesicles; M1-EVs, M1-type macrophage- derived extracellular vesicles; M2-EVs, M2-type macrophage- derived extracellular vesicles.

Aging OA fibroblast-like synoviocytes (FLSs) induce senescence in non-aging FLSs and promote the formation of an inflammatory phenotype through the secretion of EVs (47). Sequencing results found that compared with non-aging EVs, there were differential expressions of 17 miRNAs, 11 lncRNAs, 14 tRNAs, and 21 proteins in aging EVs (**Figure 4**). Bioinformatics analysis showed that these miRNAs are related to important pathways such as fibrosis, cell proliferation, autophagy, and the cell cycle; tRNAs analysis was enriched for signaling pathways including FGF, PI3K/AKT, and MAPK; while protein analysis identified upstream regulators involved in senescence and cell cycle arrest, such as PAX3-FOXO1, MYC, and TFGB1. Liu and colleagues (48) have found that the secretion of EVs from FLSs derived from OA patients was increased, and these inflammatory FLS-derived EVs (inf-EVs) promote aerobic glycolysis in macrophages, thereby facilitating the M1 polarization of macrophages. The potential mechanism is that when macrophages are stimulated by inf-EVs, the accumulation of HIF1A in macrophages increases. HIF1A is a transcription factor that plays a key role in cellular response to hypoxia and inflammatory reactions. HIF1A upregulates the expression of key enzymes in the glycolytic pathway by directly binding to the promoter regions of glycolytic genes, including glucose transporter 1 (GLUT1), hexokinase 2 (HK2), pyruvate kinase M2 (PKM2), and lactate dehydrogenase A (LDHA), activating aerobic glycolysis in macrophages. When M1-polarized macrophages are co-cultured with chondrocytes, they can inhibit the expression of proteoglycan and type II collagen in chondrocytes, and promote the expression of inflammatory factors and ECM degrading enzymes MMPs, exhibiting a phenotype similar to OA. Wang et al. found that FLSs derived EVs (FLS-EVs) highly express miR-146, which inhibits the TLR4/TRAF6/NF-κB signaling pathway by targeting TRAF6, inducing the polarization of synovial macrophages from the M1 to M2 type, and reducing cartilage damage and synovial hyperplasia (49).

### 3.2 The role of FLSs derived EVs on chondrocytes

Compared with EVs produced by unstimulated FLS, EVs from IL-1β-stimulated FLS significantly up-regulated the expression of MMP-13 and ADAMTS-5 in articular chondrocytes, and down-regulated the expression of COL2A1 and ACAN. Sequencing results showed that there were 50 miRNAs in EVs produced by IL-1β-stimulated FLS that had different expression levels compared with those from unstimulated FLS (50). Zhou and colleagues (51) found that miR-126-3p is significantly under-expressed in the synovial fluid derived EVs of OA patients, while EVs from FLSs that overexpress miR-126-3p can promote the migration and proliferation of chondrocytes, inhibit apoptosis and the expression of inflammatory factors (such as IL-1β, IL-6, and TNF-α). In the rat OA model, EVs miR-126-3p from FLS-EVs can inhibit the formation of bone spurs, prevent cartilage degradation, and exert anti-apoptotic and anti-inflammatory effects on articular cartilage. In healthy rats, miR-150-3p is enriched in circulating EVs. FLSs in healthy joints can release EVs rich in miR-150-3p, which are absorbed by chondrocytes. By activating the innate immune response, the Trim14/NF-κB/IFNβ axis is inhibited, reducing the production of pro-inflammatory cytokines in chondrocytes, and delaying the progression of OA (52). *In vivo* experiments also proved that treatment with EVs derived from healthy FLSs could suppress joint degeneration, upregulate the expression of COL II and ACAN, and downregulate the expression of innate immune-related factors such as Trim14, NF-κB, and IFN-β. miR-214-3p is low-expressed in synovial fluid EVs from OA patients. EVs secreted by FLSs with overexpressed miR-214-3p can promote the proliferation of chondrocytes, inhibit apoptosis, and reduce the levels of inflammatory cytokines such as TNF-α, IL-1β, and IL-6. In a rat model of OA, these EVs can improve the morphological changes in the synovium and cartilage tissue, inhibit the formation of osteophytes, and prevent cartilage degeneration (53). Xu found through bioinformatics analysis that miR-4738-3p is significantly low-expressed in the OA FLS-EVs. Further mechanistic studies revealed that miR-4738-3p inhibits chondrocyte inflammation and delays cartilage damage by suppressing COL2A1 expression (54).

EVs LncRNAs secreted by OA FLSs also play an important role in the occurrence and development of OA. Compared with the normal control group, the expression of PCGEM1 in the EVs derived from FLSs of OA patients is up-regulated. PCGEM1 in these EVs can up-regulate the expression of RUNX2 by adsorbing miR-142-5p, thus promoting the apoptosis of chondrocytes and the degradation of cartilage matrix. In addition, inhibiting miR-142-5p can offset the inhibitory effect of OA FLSs derived EVs PCGEM1 knockout on chondrocyte apoptosis and cartilage matrix degradation induced by IL-1β (55). Tan and colleagues (56) discovered that the expression of lncRNA H19 in the cartilage of OA patients is down-regulated, while FLSs overexpressing H19 can transfer H19 to chondrocytes through EVs, thus promoting the proliferation and migration of chondrocytes and suppressing the degradation of ECM. Specifically, FLSs derived EVs H19 can promote the expression of TIMP2 by regulating miR-106b-5p. TIMP2, which can inhibit the activity of MMPs, is crucial for maintaining the balance of the chondrocyte ECM. When H19 is overexpressed, the expression of miR-106b-5p decreases, leading to an increase in TIMP2 expression, which further reduces the expression of MMP13 and ADAMTS5 and increases the expression of COL2A1 and ACAN, inhibiting the degradation of cartilage matrix in OA.

In recent years, new forms of programmed cell death in the pathogenesis of OA have increasingly garnered the attention of researchers. Pyroptosis is a type of programmed cell death triggered by the activation of inflammasomes, characterized by severe swelling of the cell membrane and its eventual rupture, leading to the release of cellular contents and the subsequent induction of inflammatory responses (57–59). Wang and colleagues (60) found that miR-25-3p carried by FLS-EVs could enter chondrocytes, increase the expression of miR-25-3p, and simultaneously inhibit the transcription of cytoplasmic polyadenylation element-binding protein 1 (CPEB1), thereby alleviating pyroptosis of chondrocytes. The overexpression of CPEB1 reversed the inhibitory effect of FLS-EVs on pyroptosis of chondrocytes in OA. In a OA mouse model induced by destabilization of the medial meniscus, FLS-EVs can mitigate knee joint damage and symptoms, reduce the levels of MMP-3 and MMP-13, and inhibit pyroptosis of chondrocytes. Ferroptosis is a novel form of programmed cell death that is iron-dependent and distinct from apoptosis and autophagy. Its main characteristics are the accumulation of intracellular iron ions (Fe^2+^) and the excessive production of reactive oxygen species (especially lipid peroxides), leading to damage to the cell membrane, proteins, and DNA (61, 62). Research has shown that ferroptosis is closely related to the occurrence and development of OA (63, 64). Kong and colleagues (65) found that compared with the normal control group, the expression of miR-19b-3p in the EVs of OA patients’ synovial fluid was significantly up-regulated. EVs derived from OA FLSs can promote the ferroptosis of chondrocytes and reduce cell viability. The potential mechanism is that the OA FLSs derived EVs carry miR-19b-3p and deliver it to chondrocytes, targeting and inhibiting the SLC7A11 gene in chondrocytes, thus promoting the ferroptosis and damage of chondrocytes.

### 3.3 The role of synovial macrophages derived EVs in the pathogenesis of OA

Synovial macrophages are important participants in OA synovial inflammation. Synovial macrophages are plastic cells, which are divided into classically activated M1 macrophages and alternatively activated M2 macrophages. Synovial macrophages do not function alone. They engage in close communication and interaction with various cell types in the surrounding environment through paracrine or autocrine mechanisms, including synovial fibroblasts, osteoclasts, chondrocytes, lymphocytes, and adipocytes. This complex network of cellular interactions, working in concert within the joint’s internal environment, collectively drives the pathological progression of OA, exacerbating the deterioration of the disease.

The EVs released by M1-polarized macrophages (M1-EVs) can upregulate the expression of inflammatory factors and MMPs in chondrocytes, promoting inflammatory response. Through microRNA sequencing analysis, Peng and colleagues found that miR-1246 has a high expression level in M1-EVs, and miR-1246 inhibits the expression of GSK3β and Axin2 in chondrocytes by targeting them, thereby activating the Wnt/β-catenin signaling pathway and leading to inflammation in chondrocytes (66). Jia and colleagues (67) also found that M1-like macrophages can produce and release EVs containing miRNA into the joint microenvironment, which can interact with chondrocytes and affect their function. The underlying mechanism is that in an inflammatory state, M1-EVs highly express miR-146b-5p, which targets and inhibits USP3 (ubiquitin-specific protease 3) and SOX5 (SRY-related HMG-box family member 5) in chondrocytes. USP3, through its deubiquitinating enzyme activity, removes the ubiquitination of the NF-κB inhibitory protein IκB, facilitating the activation of NF-κB. SOX5, SOX6, and SOX9 can form the so-called “SOX triad” or “SOX trio,” which can inhibit the expression of cartilage formation and maintenance genes, such as COL2A1 and ACAN. Digoxin can significantly inhibit the expression of miR-146b-5p in M1-EVs, reducing the inhibitory effects on USP3 and SOX5, thereby alleviating the inflammatory and catabolic state of chondrocytes. Meanwhile, LPS-induced macrophages can secrete EVs, which can induce chondrocytes to express pyroptosis-related molecules such as NLRP3, GSDMD, IL-1β, and IL-18. The specific mechanism involves M1-EVs activating caspase-11, which then activates GSDMD, leading to the formation of pores in the cell membrane and the release of intracellular contents, causing noncanonical pyroptosis in chondrocytes. Blocking caspase 11 can alleviate the pyroptosis and catabolic processes in chondrocytes (68). Si et al. found (69) that M1-EVs highly express miR-363, and inhibit chondrocyte proliferation and survival and induce the expression of inflammatory genes through G3BP2.

M2 macrophage-derived EVs (M2-EVs) play a significant role in OA. Qian Y. et al. discovered that the expression of miR-26b-5p was notably increased in M2-EVs. miR-26b-5p is capable of repolarizing pro-inflammatory M1 macrophages to the anti-inflammatory M2 phenotype by targeting the TLR3 signaling pathway. Furthermore, miR-26b-5p can suppress the hypertrophy of chondrocytes induced by inflammation through the targeting of COL10A1. In an OA mouse model induced by the transection of the anterior cruciate ligament (ACLT), the intra-articular injection of miR-26b-5p effectively reduced synovitis and cartilage degeneration, and improved the gait abnormalities in mice (70). Yuan Q. et. al (71). and Qin L. et. al (72). also discovered similar research findings, revealing that M2-EVs can reprogram M1 macrophages into the M2 phenotype, thereby alleviating synovitis, reducing cartilage degeneration, improving subchondral bone damage, and ameliorating gait abnormalities in DMM induced mice. Yuan Q. et al. suggested that the underlying mechanisms may be associated with the NOD-like receptor (NLR), TNF, NF-κB, and TLR signaling pathways (71). Meanwhile, Qin L. et al. suggested that M2-EVs are enriched with miR-21-5p, which is a microRNA negatively associated with the degeneration of articular cartilage, capable of preventing articular cartilage damage and improving joint inflammation (72). Da-Wa ZX and colleagues administered M2-EVs via tail vein injection into a knee OA (KOA) rat model and found that M2-EVs significantly reduced the inflammatory response, decreased the levels of IL-1β, IL-6, and TNF-α in the joint tissues, improved the structure of cartilage tissue, and delayed the progression of OA (73).

## 4 The role of osteoblast and osteoclast derived EVs in the pathogenesis of OA

The subchondral bone, situated beneath the calcified cartilage, primarily consists of the subchondral bone plate and the subchondral trabecular bone (15, 74). The subchondral bone plate is a thin cortical bone layer that borders the calcified cartilage, characterized by distinct porosities housing an array of blood vessels and nerve fibers that interweave throughout. Subchondral trabecular bone is composed of cancellous bone close to the marrow cavity, which is sparse, metabolically active, and rich in blood vessels and nerves. Subchondral bone provides load absorption, structural support, and nutritional support for cartilage, and the changes in the microenvironment of subchondral bone can directly or indirectly affect cartilage metabolism (16, 74).

**Figure 5:**
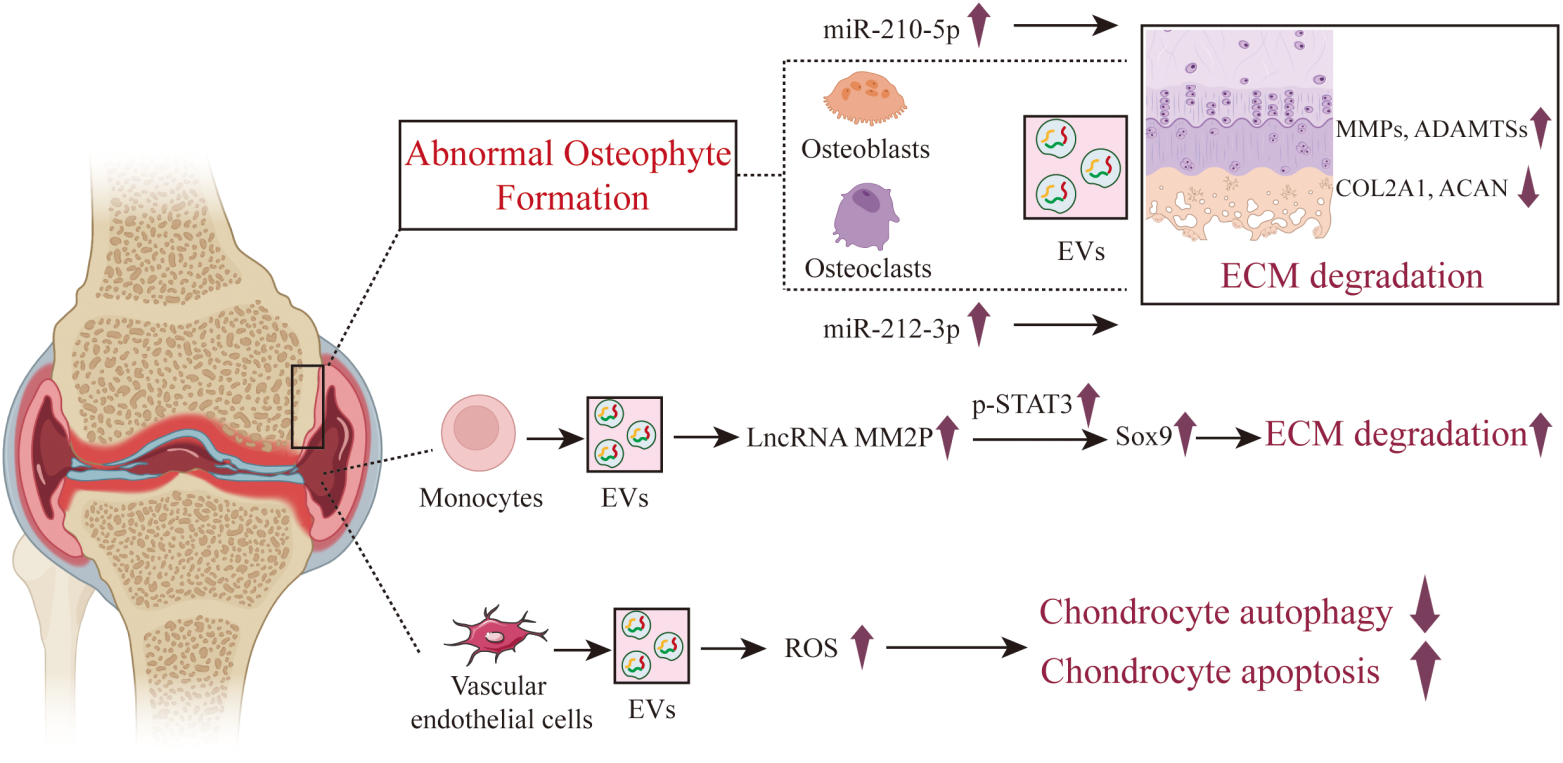
The role of osteoblast, osteoclast and other cells derived EVs in the pathogenesis of OA. In the state of OA, EVs derived from both osteoblasts and osteoclasts exhibit a role in promoting cartilage damage. The role of EVs from other intra-articular cells such as neutrophils, monocytes, and vascular endothelial cells in the course of OA is also reported, but there are relatively few articles on this subject.

Wu and colleagues found that EVs from osteoblasts of the osteoarthritic sclerotic subchondral bone are capable of being internalized by chondrocytes, leading to the upregulation of catabolic gene expression and the downregulation of chondrocyte-specific markers. The potential mechanism involves miR-210-5p, which is highly enriched in the EVs from osteoblasts of the sclerotic subchondral bone in OA, enhancing the expression of MMP13, ADAMTS5, RUNX2 and collagen type X (COL10) in particular chondrocytes, while suppressing the expression of SOX9 and COL2A1 (75) (**Figure 5**). Dai J et al. found that the conditioned medium from osteoclasts significantly suppresses the anabolism and promotes the catabolism of chondrocytes, while GW4869 markedly inhibits this effect, indicating that osteoclast-derived EVs are crucial for the impact of osteoclasts on chondrocyte function. Further mechanistic research revealed that osteoclast-derived EVs highly express miR-212-3p, which is absorbed by chondrocytes and targets the suppression of Smad2, accelerating cartilage matrix degradation in OA (76). Not come singly but in pairs, Liu and colleagues (77) discovered that compared to the sham surgery group of mice, miRNAs such as miR-21a-5p, miR-214-3p, miR-148a-3p, miR-199a-3p, miR-378a-3p, as well as miR-30, miR-200, and miR-29 were found to be significantly overexpressed in osteoclast-derived EVs from mice with OA modeled by ACLT. After the transfer of these highly expressed miRNAs from osteoclast-derived EVs to chondrocytes, the expression of tissue inhibitors of metalloproteinases-2 (TIMP-2) and TIMP-3 was suppressed, reducing the chondrocytes’ resistance to matrix degradation. TIMP-2 and TIMP-3 are endogenous inhibitors produced by chondrocytes that play a crucial role in resisting the activity of MMPs and ADAMTS family enzymes. Additionally, osteoclast-derived EVs can promote angiogenesis beneath the cartilage and affect sensory innervation by influencing the formation or activity of calcitonin gene-related peptide (CGRP) positive sensory nerve fibers.

## 5 The role of other cells derived EVs in the pathology of OA

In addition to the aforementioned cellular sources of EVs, some researchers have also studied the role of EVs from other cellular sources in the course of OA, such as monocytes, neutrophils, and other immune cell subsets, vascular endothelial cells, platelets, etc. Bai and colleagues (78) found that monocytes overexpress LncRNA MM2P after treatment with IL-4 or IL-13. MM2P is capable of blocking the dephosphorylation of STAT3 mediated by SHP2 and interacts with the RNA-binding protein FUS, thereby increasing p-STAT3 and consequently elevating the expression of SOX9. By secreting EVs, SOX9 is transferred to chondrocytes, increasing the expression of COL1A2 and ACAN, and promoting the secretion of ECM components such as proteoglycan and type II collagen, which facilitates cartilage repair. Zhan D et al. discovered that FLS internalized fluorescently labeled neutrophil-derived EVs, and healthy neutrophil-derived EVs affected the expression of proinflammatory cytokines produced by TNFα-stimulated FLSs. TNFα could stimulate the expression of cytokines in FLSs, while healthy neutrophil-derived EVs could down-regulate the expression of IL-5, IL-6, IL-8, MCP-1, IFN-γ and MIP-1β in TNFα induced FLSs (79). Kitahara et al. found that EVs from neutrophils after TGF-β intervention significantly inhibited chondrocyte degradation (80). The underlying mechanism is that TGF-β-intervened neutrophil EVs significantly promoted the upregulation of SFRP5 expression in chondrocytes, inhibiting the TNF and NF-κB signaling pathways, reducing the production of inflammatory and catabolic factors, and protecting cartilage from damage.

Yang and colleagues (81) discovered that EVs secreted by vascular endothelial cells can be absorbed by chondrocytes, increasing the ROS content of chondrocytes, exacerbating apoptosis, and inhibiting chondrocyte autophagy. Enhancing autophagy can reduce apoptosis in chondrocytes induced by IL-1β and EC-Exos, and improve the ability of chondrocytes to resist oxidative stress. Overexpression of the apoptosis-related molecule p21 can enhance the antioxidant stress ability of cells by regulating the Keap1/Nrf2/HO-1 pathway, and reduce apoptosis induced by IL-1β and vascular endothelial cells derived EVs. Furthermore, by establishing a mouse model of OA and treating with vascular endothelial cells derived EVs, the study further confirmed that vascular endothelial cells derived EVs exacerbate the progression of OA *in vivo*, reducing the ability of chondrocytes to resist oxidative stress and increasing the number of apoptotic chondrocytes.

Typically, there are no platelets in the joint cavity. When arthritis or joint injury occurs, blood may enter the joint cavity, causing platelets to enter as well. In addition, recent research has found that platelet-derived EVs (Plt-EVs) play an important role in tissue repair and regeneration, showing potential in the treatment of osteoarthritis. These EVs can be directly applied to the joint through intra-articular injection or other methods to exert their therapeutic effects. Xu and colleagues found Plt-EVs can be internalized by chondrocytes, regulating the differential expression of 1,797 genes in chondrocytes (with 923 upregulated and 874 downregulated). These genes are mainly involved in anti-inflammatory effects, cell adhesion, stimulation of cartilage repair, promotion of anabolism, and inhibition of catabolism. Meanwhile, In the ACLT mouse model, Plt-EVs significantly promoted the proliferation and migration of chondrocytes, and notably facilitated cartilage regeneration, slowing down the progression of OA (82). Liu X et al. indicates that EVs derived from Platelet-rich plasma (PRP) can promote the proliferation and migration of chondrocytes, reduce cell apoptosis, and reverse the activation of the Wnt/β-catenin signaling pathway in chondrocytes induced by IL-1β. *In vivo* experiments also show that EVs from PRP can facilitate the repair of cartilage tissue, downregulate the Osteoarthritis Research Society International (OARSI) score, and slow down the progression of OA (83).

## 6 The role of EVs in the synovial fluid in the pathology of OA

Joint cavities, as closed spaces, and synovial fluid, as the living environment for components such as the synovial membrane and cartilage within the joint, play an important role in the physiological and pathological processes of the joint. Synovial fluid is not only a medium for mutual regulation among cells within the joint cavity, but the EVs it contains are also favored by researchers for their unique role in transmitting signals between cells (84, 85). Zhao and colleagues (86) indicated that in patients with early OA and advanced OA, the expression of EVs in the synovial fluid is significantly higher than in the control group without OA. Particularly, in the synovial fluid EVs (SF-EVs) of patients with advanced OA, the expression of lncRNA PCGEM1 is markedly higher than in patients with early OA, and also significantly higher in early OA patients compared to the control group. Furthermore, the expression of lncRNA PCGEM1 in the SF-EVs can serve as a potential biomarker for distinguishing different stages of OA. Mustonen (87) focused on the expression changes of fatty acids in OA SF-EVs and found that, compared to normal SF-EVs, saturated fatty acids (such as palmitic acid, stearic acid, and behenic acid) were significantly overexpressed in OA SF-EVs. Meanwhile, Mustonen also found that higher levels of polyunsaturated fatty acids (PUFAs) and higher total dimethyl acetals in SF-EVs are closely related to knee joint function. Gao K et al. found that the levels of inflammatory cytokines (IL-1β, IL-17, IL-10, and IFN-γ) and chemokines (CCL5, CCL15, and CXCL8) in synovial fluid EVs of the advanced OA group were significantly elevated compared to the early OA group (88). Additionally, SF-EVs from the advanced OA group notably promoted chondrocyte inflammation and inhibited chondrocyte proliferation, thereby accelerating joint degeneration. Mohd Noor and colleagues found that SF-EVs induce monocytes to release the anti-inflammatory cytokine IL-1Ra, thereby regulating the immune microenvironment in OA (89).

Kolhe and colleagues (90) were the first to characterize EVs miRNA in human synovial fluid, discovering significant differences in miRNA expression in the synovial fluid EVs of OA patients compared to healthy individuals, with distinct miRNA expression patterns between males and females. The research found that the changes in miRNA expression in the synovial fluid EVs of female OA patients were more pronounced than in males. The female OA-specific miRNAs were related to estrogen responsiveness and targeted the Toll-like receptor (TLR) signaling pathways. Furthermore, when articular chondrocytes were treated with SF-EVs in OA patients, it led to increased inflammatory responses in chondrocytes and inhibited chondrocyte proliferation, thereby promoting joint degeneration. Coincidentally, Ji Y et al. found that the expression of miR-182-5p in the EVs of OA synovial fluid was significantly reduced, and the expression of miR-182-5p was negatively correlated with TNF-α (91). Overexpression of miR-182-5p can promote the proliferation and migration of chondrocytes, and it targets the suppression of tumor necrosis factor α-induced protein 8 (TNFAIP8), thereby inhibiting autophagy and apoptosis of chondrocytes through ATG3. Domeni R and colleagues (92) observed the effects of synovial fluid EVs on macrophages and found that these EVs can stimulate the polarization of macrophages towards the M1 phenotype, leading to the release of various inflammatory cytokines (such as IL-1β), chemokines (such as CCL8, CCL15, CCL20, and CXCL1), and MMPs (such as MMP1, MMP2, MMP7, MMP8, MMP10, and MMP13).

## 7 The role of joint tissue-derived EVs in the pathology of OA

EVs derived from cell lines or primary cells have garnered extensive research attention. However, the process of isolating cell populations from tissues may alter the mechanisms of EV formation and release, and these EVs often do not accurately reflect the characteristics of their original tissues. Given that EVs essentially carry information loads that are finely programmed by the cellular microenvironment, these messages are closely related to complex intercellular communication, dynamic interactions of membrane structures, and specific spatial tissue backgrounds. Tissue-derived EVs (Ti-EVs) are dispersed within the interstitial spaces of tissues and are secreted by local cell populations. These Ti-EVs more authentically provide scientific research with experimental evidence that is closer to the actual *in vivo* conditions, offering a deeper understanding of the interactions between cells and the microenvironment of the tissue (93, 94).

Chen P et al. used filtration combined with size exclusion chromatography (SECF) to extract EVs from OA synovial tissue (ST-EVs), and found that ST-EVs significantly inhibited chondrocyte proliferation and promoted apoptosis. Additionally, ST-EVs promoted the expression of pro-inflammatory cytokines and enzymes related to cartilage degradation. Moreover, ST-EVs significantly activated the NF-κB signaling pathway in chondrocytes, and the inhibition of NF-κB signaling pathway activation significantly reduced the overexpression of inflammatory cytokines and cartilage degradation-related enzymes induced by ST-EVs in chondrocytes (95). This study elucidates the impact of synovial inflammation on cartilage in the context of OA from the perspective of tissue EVs. Wu and colleagues (96) found that miR-182 was significantly overexpressed in the EVs of OA synovial tissue, and miR-182 regulates the expression of inflammatory factors (IL-6, IL-1β, and TNF-α) by suppressing the expression levels of FOXO3. Cao Y and colleagues (97) were the first to observe the effects of EVs derived from the infrapatellar fat pad (IPFP) on cartilage metabolism and cellular senescence. The study found that the IPFP from patients with OA could secrete EVs and deliver them to chondrocytes, promoting the degradation of the ECM and inducing chondrocyte senescence. The use of GW4869, an inhibitor of EVs release, significantly mitigated the cartilage destruction induced by IPFP-EVs. Mechanistically, the high content of let-7b-5p and let-7c-5p in IPFP-EVs targets and suppresses the expression of lamin B receptor (LBR), while the inhibition of let-7b-5p and let-7c-5p can increase the expression of LBR, inhibit chondrocyte senescence, and improve the progression of OA.

## 8 Conclusions and prospects

With the continuous expansion and deepening of research in the field of OA, the exploration of intercellular communication mechanisms within joints, particularly how signals are precisely transmitted within these intricate cellular networks, has emerged as a major research focus. Since Stahl and Johnstone first discovered EVs in immature red blood cells more than forty years ago, this discovery has opened a new era of intercellular communication, prompting a surge in related research. EVs, as “messengers” of intercellular information transmission, can carry a variety of bioactive molecules, such as proteins, lipids, and non-coding RNA, shuttling between cells to achieve precise biological signal transduction functions, and their potential and mysteries have fascinated researchers. Leveraging advanced “omics” technologies, researchers are now able to delve into the contents of EVs, particularly ncRNA molecules with regulatory potential, providing a powerful tool for unraveling the intercellular communication mechanisms underlying OA pathology. In OA research, ample evidence suggests that EVs secreted by various cells within joints, particularly chondrocytes, macrophages, and synovial fibroblasts, play pivotal roles in the onset and progression of OA. These EVs not only participate in regulating the homeostasis of the joint microenvironment but also directly impact the pathological processes of OA. However, given that OA is a systemic joint disease, its pathological characteristics are characterized by abnormal regulation of gene expression through multiple pathways and levels, and although the study of EVs from a single cell type is insightful, it is difficult to fully reflect the complex biological processes of OA in the body. Consequently, studies on EVs derived from different tissues have gradually gained traction, offering a novel perspective for exploring the pathological mechanisms of OA.

This article aims to provide a comprehensive review of the mechanisms of action of EVs from different cells and tissues in OA, and to deeply analyze how these EVs shuttle between cells and tissues during the course of OA, transmitting inflammatory signals that drive the progression of the disease. The article also explores whether intervention in the pathways of these inflammatory signals could potentially halt or even reverse the course of OA, paving the way for new therapeutic strategies and offering hope for the future management of the condition. This research direction not only helps to deepen our understanding of the pathological mechanisms of OA but also provides valuable theoretical foundations and practical directions for the development of new therapeutic approaches and the improvement of patients’ quality of life.
