# Supplementary material for: The communication role of extracellular vesicles in the osteoarthritis microenvironment
Source: Front Immunol. 2025 Mar 17;16:1549833. doi: 10.3389/fimmu.2025.1549833 (PMC11955493; doi:10.3389/fimmu.2025.1549833)
Supplement: Supplementary file 1 [file Table1.docx]

**The search strategy**

1. Extracellular vesicles [Title/Abstract]

2. Exosomes [Title/Abstract]

3. Exosome* [Title/Abstract]

4. Microvesicles [Title/Abstract]

5. Exovesicle* [Title/Abstract]

6. 1 OR 2 OR 3 OR 4 OR 5

7. Osteoarthritis [Title/Abstract]

8. Osteoarthritides [Title/Abstract]

9. Osteoarthrosis [Title/Abstract]

10. Osteoarthroses [Title/Abstract]

11. Arthritis [Title/Abstract]

12. Arthritides [Title/Abstract]

13. Arthrosis [Title/Abstract]

14. Arthroses [Title/Abstract]

15. 7 OR 8 OR 9 OR 10 OR 11 OR 12 OR 13 OR 14

16 6 AND 15
